# Elastic Bands Improve Oral Appliance Treatment Effect on Obstructive Sleep Apnoea: A Randomised Crossover Trial

**DOI:** 10.1111/joor.13870

**Published:** 2024-09-30

**Authors:** Ulrik Leidland Opsahl, Morten Berge, Sverre Lehmann, Bjørn Bjorvatn, Anders Johansson

**Affiliations:** ^1^ Department of Clinical Dentistry, Faculty of Medicine Center for Translational Oral Research (TOR), University of Bergen Bergen Norway; ^2^ Norwegian Competence Center for Sleep Disorders Haukeland University Hospital Bergen Norway; ^3^ Department of Clinical Science, Faculty of Medicine University of Bergen Bergen Norway; ^4^ Department of Thoracic Medicine Haukeland University Hospital Bergen Norway; ^5^ Department of Global Public Health and Primary Care University of Bergen Bergen Norway

**Keywords:** monobloc, mouth opening, oral appliance design, positional OSA, prediction, randomised

## Abstract

**Background:**

Oral appliances (OAs) that limit mouth opening during sleep, such as monobloc appliances, have shown superior treatment effects in subgroups of patients with obstructive sleep apnoea. The application of elastic bands on bibloc appliances may resemble these benefits.

**Objectives:**

The primary objective was to investigate if application of elastic bands to bibloc appliances improves treatment success (> 50% reduction of respiratory event index (REI)), in addition to other subjective variables. Furthermore, we aimed to identify variables predicting the need for elastic bands in OA treatment.

**Methods:**

Included patients (*n* = 69) were randomly assigned to OA treatment with or without elastic bands. After 3 weeks, treatment effect was investigated with home respiratory polygraphy and questionnaires. Thereafter, patients changed treatment modality, with identical follow‐up regime. Statistical analyses were performed using Student's *t*‐test and Pearson's chi‐squared test to investigate differences between the two treatment modalities, and logistic regression analysis was conducted to investigate variables tentatively associated with treatment success.

**Results:**

Based on REI, the success rate with OA treatment was in favour of elastic bands (53.9% vs. 34.6%, *p* = 0.002). Male sex and larger maximum mouth opening were identified as predictors for increased treatment success with elastic bands. The main benefit with elastic bands seemed to be greater reduction of REI when supine. However, patients seem to prefer OA without elastic bands.

**Conclusions:**

Elastic bands improved OA treatment effect by reducing the REI in supine position. Patient groups that seemed to benefit from elastic bands in OA treatment were men with large maximum mouth openings.

## Graphical abstract

Does elastic bands improve oral appliance (OA) treatment success of obstructive sleep apnoea (OSA)? Yes it does.

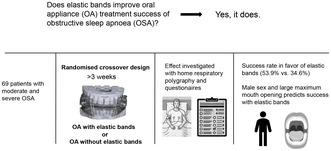

## 1 Background

Obstructive sleep apnoea (OSA) is characterised by frequent collapse of the upper airway during sleep, causing hypoxia, hypercapnia and sleep fragmentation [1]. Consequences of OSA, if left untreated, may include excessive daytime sleepiness, impaired quality of life (QoL) and comorbid cardiovascular conditions [1]. It has been estimated that approximately 425 million adults aged 30–65 suffer from moderate to severe OSA globally [2].

Positive airway pressure (PAP) is generally considered the first‐line treatment option for OSA [3]. Even though PAP commonly achieves optimal treatment effects, the main challenge for PAP is treatment adherence [4, 5, 6, 7]. Oral appliance (OA) treatment is often the go‐to treatment for patients without adherence to PAP. Although OA treatment generally is regarded effective [8], as many as 25%–45% may be considered failures, defined as ≤ 50% reduction in baseline respiratory event index (REI). Hence, the need to improve OA treatment effect is evident.

OAs come with different designs. Bibloc appliances are generally considered the gold standard due to better treatment effect [9] and adherence [10], compared to monobloc appliances. However, some studies have shown that patients may benefit from OAs that limit mouth opening during sleep by using monobloc appliances [11, 12]. The application of elastic bands on bibloc appliances may limit mouth opening during sleep, and in many ways transform a bibloc appliance to a monobloc appliance. A small randomised pilot study with crossover design investigated application of elastic bands in OA treatment and showed higher success rate with elastic bands compared to without (90% success vs. 70% success). The greatest differences were shown in two patients with positional‐dependent OSA (POSA) [13].

Cartwright defined POSA as REI supine > 2 × REI nonsupine [14]. The prevalence of POSA in a previous study was found to be 18%–34% [15]. Elastic bands application in OA therapy in patients with POSA has shown to significantly increase success rates (> 75% reduction of REI) compared to OA without elastic bands (67.5% vs. 36.2%) [16]. It is hypothesised that patients treated with bibloc appliances without connectors (e.g., SomnoDent Fusion, SomnoMed Ltd.) will benefit from using elastic bands, mainly due to prevention of opening of the mouth and mandibular retrusion during sleep in supine position. In mouth opening, the mandible retrudes together with the tongue and the soft palate, which may lead to decreased upper airway volume [17, 18], and thus, reduced effect of the OA. Consequently, patients with POSA are hypothesised to benefit the most from applying elastic bands when treated with OA.

Generally, there is a lack of evidence regarding application of elastic bands in OA treatment, and above‐mentioned studies have several limitations, with limited number of patients [13] or weak study design [16]. Thus, there is a need for randomised controlled trials with appropriate sample sizes to investigate the effect of elastic bands in OA treatment. The primary objective of this study was to investigate if elastic bands improve OA treatment success based on > 50% reduction in REI. A further aim was to explore if there were differences regarding adherence, side effects and subjective effects between the two treatment modalities and if investigated variables can predict the need for elastic bands in OA treatment.

## 2 Methods

The study was designed as a single‐centre, randomised crossover trial of patients with OSA.

Eligible patients were men and women aged 18 years and older referred for treatment of moderate or severe OSA. All patients were nonadherent to PAP treatment and were thus referred for OA treatment. Eligible patients participated in the ‘Sleep Registry’ at the Center for Sleep Medicine, Haukeland University Hospital. Patients were excluded if they had mild or no OSA, had insufficient number of teeth to retain an OA, had complete dentures, did not speak and/or read the Norwegian language and were not capable of giving informed consent.

All eligible patients were in a consecutive order invited to participate in the trial until the estimated number of patients was included. All parts of the treatment took place at the Center for Sleep Medicine, Haukeland University Hospital.

### 2.1 Device

At the Center for Sleep Medicine, the ‘Narval CC’ appliance (ResMed) is prioritised as the primary choice for OA treatment, decided by a tender. The indication for treatment with the ‘SomnoDent Fusion’ appliance (SomnoMed) at the Center for Sleep Medicine is insufficient retention on remaining teeth to support a ‘Narval CC’ appliance (ResMed). Eligible patients were all considered for treatment with ‘Somnodent Fusion’ but were excluded if they were suitable for treatment with the ‘Narval CC’ appliance.

Maximal protrusion was measured using the George Gauge bite fork, and the increase of occlusal vertical dimension was reduced to the minimal height required for the ‘SomnoDent Fusion’ appliance (4–5 mm). The OAs were produced in 63% or 69% of maximal protrusion for patients with moderate and severe OSA, respectively. These positions have been reported as being optimal for the mandible following stepwise, objective titration [19].

**FIGURE 1:**
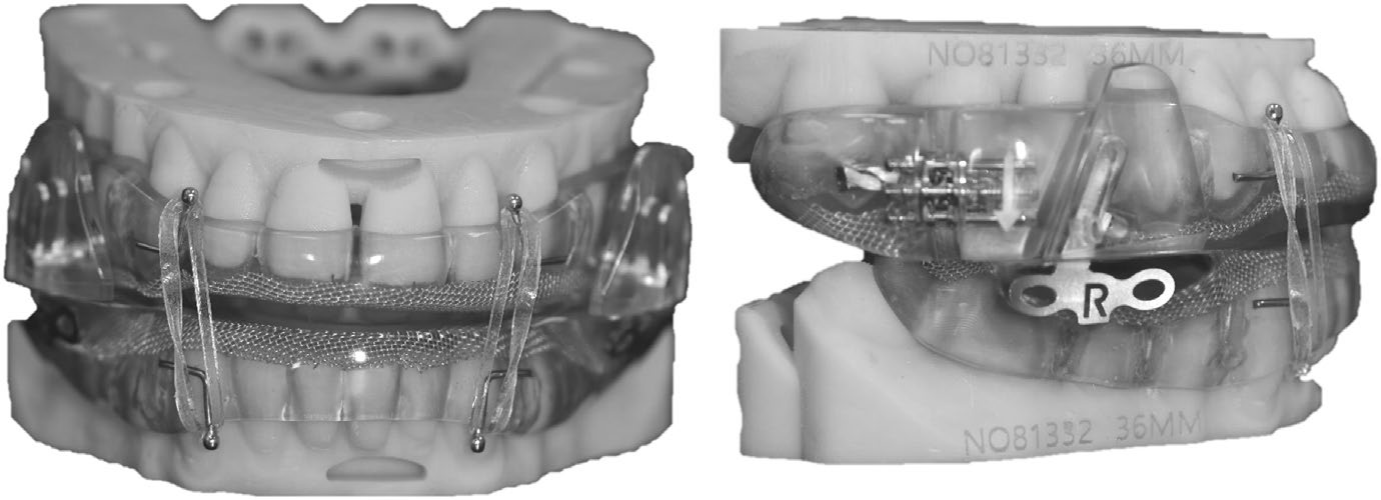
The oral appliance applied in the study with elastic bands.

Elastic bands were attached to anteriorly placed hooks on both the upper and lower appliance, bilaterally (see Figure 1). The elastic bands were carefully selected so as not to interfere with retention of the OA. The strongest elastic bands that did not interfere with retention of the OA were selected, within the range of 85–170 g (3/8″–3/16″).

### 2.2 Randomisation Sequence

Patients were randomised to initiate treatment either with or without elastic bands with simple random allocation, by drawing one of two identical balls labelled ‘with elastic bands’ or ‘without elastic bands’. The patients drew the balls from a container without visual access to the balls in the drawing process. Drawing was performed initially at the first visit of the trial, subsequent to an oral repetition of study details with the patients.

Simple randomisation method was selected based on the assumption that there was no sequence effect within the study [20]. Therefore, it is presumed that imbalance in the treatment sequence has minimal effect on the results [21].

### 2.3 Intervention

Participants were treated with OA, both with and without elastic bands for 3 weeks. The effect of the treatment was investigated with home respiratory polygraphy registrations (PG) with Type IV devices (Nox T3, Nox Medical), including manual scoring, and questionnaires at the end of each 3‐week period. Thereafter, patients changed treatment modality (with or without elastic bands), with identical assessment of treatment effect after 3 weeks of treatment.

After both treatment modalities were tested, patients were informed of the results from the PG recordings, and patients chose their preferred modality for further treatment. Treating dentist advised the patients to choose the treatment modality resulting in the largest REI reduction. Success was categorised into four criteria from Gjerde et al. [22] based on the reduction in REI following OA treatment: Success criterion 1 was defined as REI < 5 postop, criterion 2 was defined as REI < 10 + REI reduction > 50% from baseline, criterion 3 as REI reduction > 50% from baseline and criterion 4, also termed treatment failure, as REI reduction ≤ 50% from baseline.

If a patient accomplished Success Criterion 1 or 2 with the chosen treatment modality after the intervention, no titration was performed. In the event of Success Criterion 3 or treatment failure with the chosen modality, the OA was titrated stepwise by 1 mm incrementation to locate the optimal position. Every titration was controlled using PG recordings to examine its impact.

For patients not able to use their OA due to side effects at any point during the 3‐week period, the issues causing discomfort were handled adequately, and control of the treatment with PG and questionnaires were postponed for 2 weeks if remaining time until the planned control was less than 2 weeks. Patients were encouraged to make contact if side effects/discomfort related to the treatment occurred. If a patient was unable to complete the intervention protocol due to side effects/discomfort, the treatment was changed to the other treatment modality (i.e., from OA with elastic bands to OA without elastic bands), and the OA was titrated optimally. If a patient was unable to use any of the treatment modalities, and thus is nonadherent to OA treatment, PAP treatment was reoffered. Similarly, if a patient was deemed as nonresponder to OA treatment after both interventions, and further titration to optimise the OA proves ineffective, the patient was reoffered PAP treatment.

### 2.4 Outcomes

The primary outcome measure of this study was to investigate differences in success, defined as > 50% reduction of REI, comparing OA treatment with and without elastic bands. The secondary outcome measures included assessing differences in the subjective effect of OA treatment with and without elastic bands through questionnaires. These questionnaires aimed to explore variables regarding treatment adherence, side effects, quality of life, partner perception of the treatment, insomnia, snoring, daytime somnolence and anxiety/depression.

### 2.5 Measurements

#### 2.5.1 Respiratory Polygraphy Registrations

The PG recordings were manually scored by the same clinician blinded to the treatment modality (with or without elastic bands) using criteria in accordance with the American Academy of Sleep Medicine 2012 guidelines [23].

#### 2.5.2 Questionnaires

A questionnaire used in all patients referred to the sleep apnoea clinic at the Centre for Sleep Medicine, Haukeland University Hospital, is included in the local ‘Sleep Registry’ and applied in this study. This contains questions regarding the patient's history of sleep disorders and the patient's sleep habits, subjective evaluated health, QoL, experienced snoring, breathing cessations and daytime somnolence during the past 3 months, Epworth Sleepiness Scale (ESS), questions regarding subjective nasal congestion, Bergen Insomnia Scale (BIS), questions regarding restless legs and circadian rhythm, the Hospital Anxiety and Depression Scale (HADS), questions regarding smoking, alcohol and coffee consumption and the Fatigue Severity Scale (FSS 7‐item).

ESS is used for measuring daytime sleepiness through eight questions regarding the propensity to fall asleep in different everyday situations. Each question is scored from 0 to 3, with a total score based on a scale of 0–24. Scores > 10 indicate excessive daytime sleepiness [24].

BIS is a validated scale for measuring insomnia consisting of six items scored on an 8‐point scale (0–7) [25]. Chronic insomnia is defined as scoring ≥ 3 in one or more of Items 1–3 in addition to scoring ≥ 3 in one or more of Items 5–6.

HADS is a questionnaire containing 14 questions that can be divided into two subquestionnaires regarding anxiety (HADS‐A) and depression (HADS‐D). The intention of HADS is to identify patients with high possibility and probability of anxiety and/or depression in a nonpsychiatric hospital clinic [26]. HADS‐A and HADS‐D scores between 0 and 7 are considered asymptomatic, and scores > 7 indicate symptoms of anxiety and depression, respectively.

FSS 7‐item measures subjective fatigue through seven questions scored on a 7‐point Likert scale (1 = strongly disagree, 7 = strongly agree) [27]. FSS score was reported as the mean score of the seven questions, and scoring of high fatigue is defined as mean FSS score ≥ 5 [28].

In addition, the questionnaire contained questions about whether the patients previously had been diagnosed with myocardial infarction, stroke, diabetes, hypertension, chronic obstructive pulmonary disease, asthma, angina pectoris and/or depression.

At the end of both 3‐week treatment periods, the patients answered an additional questionnaire regarding reported adherence to treatment, subjective effects and side effects. Regarding treatment adherence, patients were asked two questions: The first question was ‘On average, how many nights per week do you use your oral appliance’, with five response options: ‘0 nights/week’, ‘1–2 nights/week’, ‘3–4 nights/week’, ‘5–6 nights/week’ and ‘7 nights/week’. The second question was ‘On average, how many hours do you use your oral appliance per night’, with alternatives: ‘0–2 h/night’, ‘2–3 h/night’, ‘3–4 h/night’, ‘4–5 h/night’ or ‘> 5 h/night’.

Concerning subjective effect, patients were asked ‘how is your daytime sleepiness affected by the oral appliance treatment’. Response options were ‘increased daytime sleepiness’, ‘unchanged’, ‘reduced daytime sleepiness’ and ‘complete relief of daytime sleepiness’. Furthermore, patients were asked ‘how is your quality of life affected by the oral appliance treatment’, with response options ‘worsening’, ‘unchanged’ and ‘improved’.

Regarding side effects, patients were asked to check boxes in the questionnaire if they had experienced any of the following side effects when using their oral appliance: ‘temporomandibular joint pain’, ‘headache’, ‘pain in masticatory muscles’, ‘increased salivation’, ‘dry mouth’ and ‘occlusal changes’.

### 2.6 Sample Size Calculation

Prior to the study, sample size calculation was performed with 5% level of significance and 80% power, using data from above‐mentioned pilot study with similar study design [13], where treatment success (> 50% reduction of REI) with and without elastic bands was 90% and 70%, respectively. The result of this calculation determined that 124 patients (62 per group) were sufficient for this study. Being a crossover trial, this would correspond to 62 patients in total. To account for 10% attrition rate, 69 patients were recruited in total. Sample size calculations were performed using the statistical software ‘Stata 18’ (StataCorp LLC) [29].

### 2.7 Statistics

All statistical analyses were performed using Stata 18 (StataCorp LLC). Student's *t*‐test was performed to investigate differences between continuous variables following OA treatment with and without elastic bands, and Pearson's chi‐squared test was performed to investigate differences between categorical variables posttreatment. Values pre‐ and posttreatment were used to investigate effect within each treatment modality (with and without elastic bands), and delta values (change pre‐ to posttreatment per treatment modality) were used to investigate differences between the two treatment modalities.

Logistic regression analysis was performed to investigate variables predicting treatment success exclusively with OA treatment with elastic bands, with ‘treatment success exclusively with elastic bands’ (yes or no) as a dependent, bivariate variable. Selection of independent variables for the analysis encompassed variables that theoretically could influence treatment outcomes based on previous research on prediction of responders to OA treatment [30, 31]. Patients who were nonadherent to OA treatment with or without elastic bands were excluded from the analysis. To adjust for the repeated design (crossover), we applied logistic regressions with cluster robust variance estimates for two observations per individual when investigating differences in success with elastic bands between men and women.

Differences in side effects between the two treatment modalities were investigated using binomial probability tests, investigating the probability of observing identical or higher frequency of side effects with elastic bands compared to OA treatment without elastic bands. In addition, binomial probability tests were used to investigate differences in frequency of ESS, BIS, HADS‐A, HADS‐D and FSS between the two treatment modalities.

Student's *t*‐test was performed to investigate differences between patients completing both interventions and patients dropping out from the study for the variables age, gender, BMI, neck circumference, REI baseline, REI supine baseline, SaO_2_, SaO_2_ nadir, overjet, overbite and maximum mouth opening. The level of significance was set to *p* < 0.05 for all above‐mentioned statistical tests.

### 2.8 Ethics

The trial was approved by the Regional Ethics Committee of Western Norway (Protocol No: 550079 REK Vest). The study was also approved by the health and social services representative of both the University of Bergen and Haukeland University Hospital. Written informed consent was obtained by all participants before treatment started. The study was registered at clinicaltrials.gov prior to trial start (ID: NCT05987618).

## 3 Results

**FIGURE 2:**
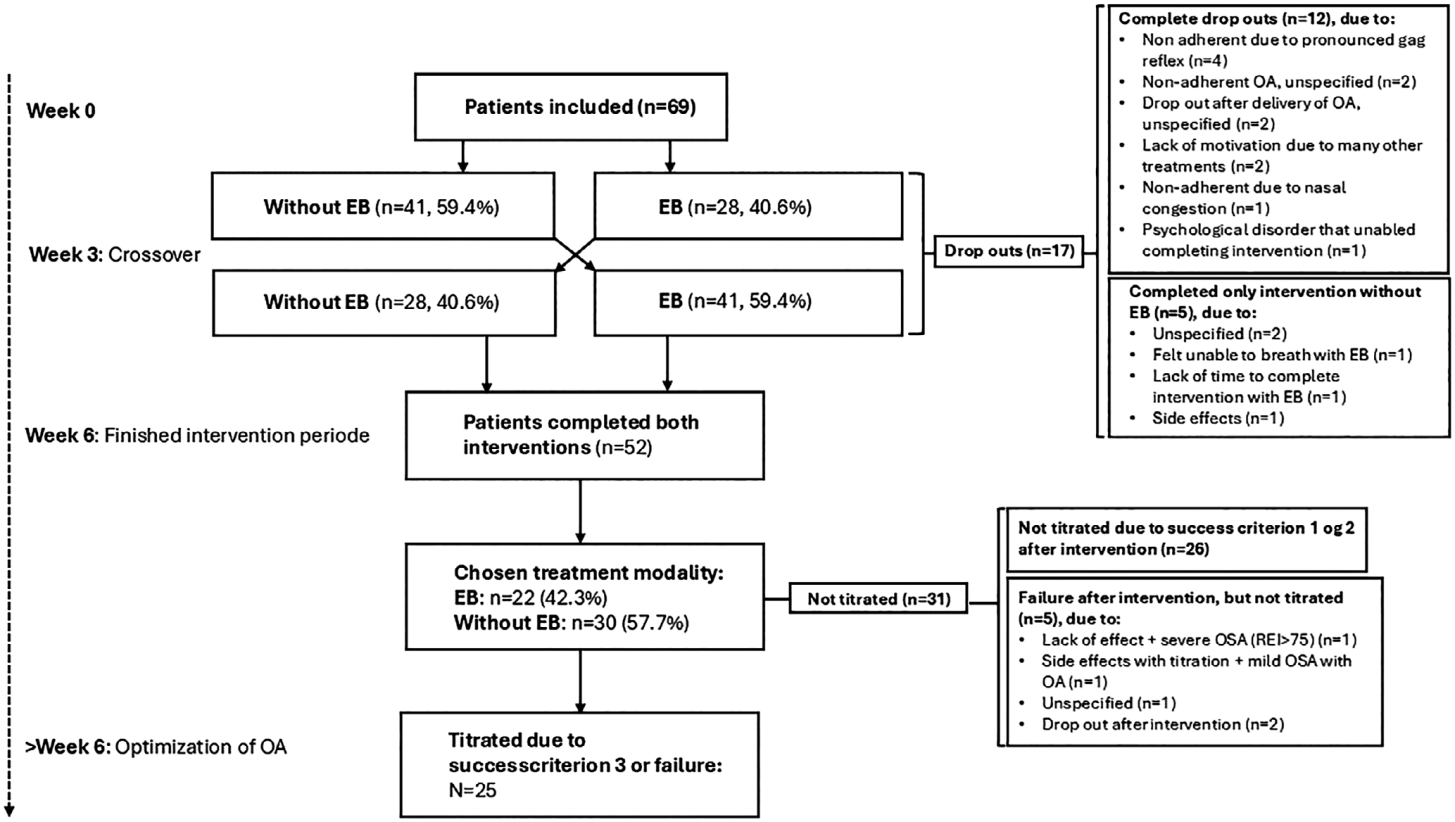
Study flow chart. EB = elastic bands, OA = oral appliance, REI = respiratory event index.

A total of 69 patients were included in the study (19 females and 50 males). In total, 52 patients completed both arms of the trial (16 females and 36 males). A chart for a detailed description of the participants’ study flow is shown in Figure 2.

Their baseline characteristics are described in Table 1. All included patients (*n* = 52) were noncompliant with PAP therapy, and six patients (11.5%) had previously undergone surgical treatment for OSA/snoring indication. Among included patients, 23 (45.1%) were diagnosed with hypertension, five (9.6%) with diabetes, seven (13.5%) with asthma, three (5.8%) with chronic obstructive pulmonary disease, one (1.9%) with angina pectoris and one (1.9%) with a history of myocardial infarction. None had previously suffered from a stroke, and 10 (19.2%) patients reported depression at baseline.

**TABLE 1:** Average baseline characteristics of patients who completed both interventions (*n* = 52).

Ref. category	Patients	Mean	SD	Min, max
Age, years	52	52.9	14.6	21–0, 80.0
BMI	52	31.1	5.9	21.6, 50.5
Neck circumference, cm	51	41.5	3.5	34.0, 52.0
REI	52	30.3	13.1	15.2, 76.3
REI supine	47	41.4	17.2	19.0, 83.6
REI nonsupine	36	22.1	14.9	1.8, 69.3
ODI	52	27.5	12.7	10.7, 75.9
AI	45	13.5	14.3	0.3, 74.1
HI	45	16.8	7.6	2.1, 37.5
Snore frequency, %	40	27.3	20.2	0.0, 86.4
SaO_2_ mean, %	52	92.5	1.8	84.5, 94.7
SaO_2_ nadir, %	51	77.6	7.6	52.0, 89.0
Registration time < 90% SaO_2_, %	51	10.7	16.6	0.0, 90.3
Overjet, mm	52	2.5	1.3	0.0, 5.0
Overbite, mm	52	3.0	1.8	0.0, 7.0
Maximum mouth opening, mm	52	51.2	6.3	36.0, 65.0
Maximum protrusion, mm	52	8.8	2.3	3.0, 14.0
Distance IP‐RP, mm	52	2.4	1.0	1.0, 4.5
CPAP pressure maximum, cmNote H_2_O	33	10.9	3.4	6.3, 19.9
CPAP pressure median, cm H_2_O	34	7.4	2.7	4.2, 14.1
CPAP pressure 95th percentile, cm H_2_O	35	10.0	3.2	5.3, 18.4
Epworth Sleepiness Scale	52	7.9	4.3	0.0, 17.0
Excessive daytime sleepiness	16 (30.8%)			
Bergen Insomnia Scale	52	16.0	9.2	0.0, 38.0
Chronic insomnia	26 (50.0%)			
Anxiety, HADS	52	5.6	4.1	0.0, 14.0
Symptoms of anxiety	14 (26.9%)			
Depression, HADS	52	4.4	3.6	0.0, 14.0
Symptoms of depression	13 (25.0%)			
Fatigue Severity Scale, mean	52	3.5	1.7	1.0, 7.0
High fatigue	13 (25.0%)			

*Note:* Excessive daytime sleepiness is defined as ESS > 10. Chronic insomnia is defined as scoring ≥ 3 in one or more of Items 1–3 on Bergen Insomnia Scale, in addition to scoring ≥ 3 in one or more of Items 5–6 on Bergen Insomnia Scale. Symptoms of anxiety and depression are scored as HADS‐A and HADS‐D > 7, respectively. High fatigue is defined as scoring Fatigue Severity Scale ≥ 5.

Abbreviations: AI = apnoea index, BMI = body mass index, Diff. = significantly different, HADS = Hospital Anxiety and Depression Scale, HI = hypopnea index, IP = intercuspidal position, ODI = oxygen desaturation index, OSA = obstructive sleep apnoea, REI = respiratory event index, RP = retruded position, SD = standard deviation.

The average protrusion of the OAs was 5.7 mm, ranging from 2.1 to 9.6 mm. The average thickness of the OAs, meaning the interincisal distance with the OA in situ, was 5.4 mm, ranging from 4 to 8 mm. When including the vertical overbite of the patients, the average increase of the total vertical opening with the OA in situ was 8.4 mm, ranging from 4 to 12 mm.

### 3.1 Comparison Between the Treatment Modalities

#### 3.1.1 Objective Measured Results of the Intervention

The success rates (> 50% reduction of REI) with OA treatment with and without elastic bands were significantly in favour of treatment with elastic bands (53.9% vs. 34.6% success, *p* = 0.002). Results from PG registrations, displaying outcomes of both interventions are further described in Table 2.

**TABLE 2:** Effects of oral appliance treatment with and without elastic bands on objectively measured variables for patients who completed both interventions (*n* = 52).

		With elastic bands			Without elastic bands			
Ref. category	*n*	Mean (SD) pre–post	Diff.	*p*	Mean (SD) pre–post	Diff.	*p*	*p* diff. Δ
REI	52	30.3 (13.1)–18.1 (15.0)	12.2	**< 0.001**	30.3 (13.1)–20.0 (16.5)	10.3	**< 0.001**	NS
REI supine	47	41.4 (17.2)–23.2 (19.0)	18.2	**< 0.001**	41.4 (17.2)–27.6 (21.4)	13.8	**< 0.001**	**0.041**
REI nonsupine	36	22.1 (14.9)–14.8 (14.0)	7.3	**< 0.001**	22.1 (14.9)–15.8 (14.3)	6.3	**0.002**	NS
PG time supine, %	43	47.0 (24.0)–49.4 (25.4)	−2.4	NS	47.0 (24.0)–41.0 (24.4)	6.0	NS	**< 0.001**
SaO_2_ mean, %	52	92.5 (1.8)–92.4 (2.1)	0.1	NS	92.5 (1.8)–92.6 (2.0)	−0.1	NS	NS
SaO_2_ nadir, %	51	77.6 (7.6)–82.1 (7.8)	−4.5	**0.001**	77.6 (7.6)–82.0 (7.0)	−4.4	**< 0.001**	NS
Registration time < 90% SaO_2_, %	51	10.7 (16.6)–9.6 (15.1)	1.1	NS	10.7 (16.6)–10.8 (17.0)	−0.1	NS	NS

*Note:* Differences were investigated using Student's *t*‐test, both pre–postdifferences and differences in delta values between the two treatments. The bold values are all *p*‐values below the level of significance ( < 0.05).

Abbreviations: diff Δ = differences in delta values pre–posttreatment with and without elastic bands, diff = difference pre‐ and posttreatment, *n* = number of patients, NS = no statistically significant differences, PG = home respiratory polygraphy registrations, REI = respiratory event index, SD = standard deviation.

#### 3.1.2 Subjective Measured Results of the Intervention

The frequency of reporting ‘improved quality of life’ after treatment was significantly higher using OA without compared to elastic bands (71.1% vs. 56.3%, *p* = 0.018). Daytime sleepiness (ESS) was reduced after OA treatment compared to baseline values, both with (7.9 to 6.2, *p* < 0.001) and without (7.9 to 6.6, *p* < 0.002) elastic bands, without significant differences between the two treatment modalities. However, the frequency of participants with excessive daytime sleepiness (ESS > 10) was only significantly reduced without elastic bands, but not with elastic bands (Table 3). A total of 66.7% and 69.6% with and without elastic bands, respectively, reported reduced daytime sleepiness on the question ‘how is your daytime sleepiness affected by the oral appliance treatment’. Complete relief of daytime sleepiness was reported by 7.8% (with elastic bands) and 8.7% (without elastic bands) on the same question. These results did not differ between treatment modalities.

**TABLE 3:** Reported percentage reduction in subjectively reported conditions with and without elastic bands, pre‐ and postoral appliance treatment, for patients who completed both interventions (*n* = 52).

	With elastic bands			Without elastic bands			
Ref. category	Mean pre–post, %	Diff.	*p*	Mean pre–post, %	Diff.	*p*	*p* diff. Δ
Excessive daytime sleepiness (ESS)	30.8–20.0	10.8	NS	30.8–16.7	14,1	**0.041**	NS
Chronic insomnia (BIS)	50.0–47.9	2.1	NS	50.0–44.7	5.3	NS	NS
Anxiety (HADS‐A)	26.9–23.4	3.5	NS	26.9–32.6	−5,7	NS	NS
Depression (HADS‐D)	25.0–15.2	9.8	NS	25.0–17.0	8.0	NS	NS
High fatigue (FSS 7‐item)	25.0–14.3	10.7	NS	25.0–19.2	5.8	NS	NS

*Note:* Differences were investigated using two‐sided binomial probability tests. The bold values are all *p*‐values below the level of significance ( < 0.05).

Abbreviations: BIS = Bergen Insomnia Scale, diff. Δ = differences in delta values pre–posttreatment with and without elastic bands, Diff. = difference in percentage points pre‐ and posttreatment, ESS = Epworth Sleepiness Scale, FSS 7‐item = Fatigue Severity Scale, HADS = Hospital Anxiety and Depression Scale.

No significant differences were observed in reported chronic insomnia (based on BIS), anxiety (HADS‐A), depression (HADS‐D) or fatigue (FSS 7‐item) following both interventions (Table 3).

#### 3.1.3 Reported Adherence to the Treatment

A total of 91.8% of the patients reported using their OA with elastic bands 5 nights or more per week compared to 95.9% reported usage without elastic bands (*p* = 0.01). Overall, 94.5% reported usage of their OA with elastic bands 4 h or more per night compared to 98.6% without elastic bands (NS).

#### 3.1.4 Side Effects

No statistically significant differences were observed in reported side effects between the two treatment modalities: Temporomandibular joint pain occurring within the treatment period was reported by 16.7% and 15.2% with and without elastic bands, respectively. The corresponding figures for headache were 8.3% and 6.5%, pain in masticatory muscles 8.3% and 6.5%, increased salivation 25.0% and 23.9%, dry mouth 22.9% and 19.6% and occlusal changes 18.8% and 13.0%.

#### 3.1.5 Predictive Factors for Superior Effect Using Elastic Bands

After patients had completed treatment with both interventions, 13 (25.0%) patients had been successfully treated exclusively with elastic bands. The remaining 39 patients (75%) that completed both interventions were either successfully treated both with and without elastic bands (28.8%), successfully treated exclusively without elastic bands (5.8%) or failed with both treatment modalities (40.4%). Of the 13 patients successfully treated exclusively with elastic bands, 11 (84.6%) were men and 2 (15.4%) were women.

Logistic regression showed that a significantly greater chance of reducing REI > 50% from baseline with elastic bands was observed for men (OR:2.9, 95% CI: 1.35–6.21, *p* = 0.006) but not for women (OR: 1.30, 95% CI: 0.53–3.20, NS). In addition, maximum mouth opening at baseline was significantly larger in the group exclusively successful with elastic bands, with 54.5 mm (SD:6.0 mm) maximum mouth opening, compared to 50.1 mm (SD:6.0 mm) in the remaining patients (OR:1.13, 95% CI: 1.01–1.27, *p* = 0.035).

#### 3.1.6 Subgroup Analysis

Subgroup analysis was performed for the group of patients predicted to be responders to OA treatment with elastic bands, which were men with maximum mouth opening above 50 mm (50th percentile). The subjective variables that showed significant differences in favour of OA treatment without elastic bands were investigated for this subgroup of patients (frequency of reporting ‘improved quality of life’, frequency of participants with excessive daytime sleepiness (ESS > 10) and higher frequency of reported usage ≥ 5 nights/week). No significant differences were shown for any of the investigated variables for men with maximum mouth opening above 50 mm in the study population that completed both interventions.

#### 3.1.7 Missing Data Analysis

Of the included 69 patients, a total of 17 (24.6%) dropped out during the intervention for various reasons (Figure 2). In missing data analyses, no significant differences were observed between study drop‐outs (*n* = 17) and completers (*n* = 52) for the investigated variables age (52.9 years vs. 50.8 years), gender (30.8% females vs. 17.7% females), BMI (31.1 vs. 31.7), neck circumference (41.5 cm vs. 42.9 cm), REI baseline (30.3 vs. 34.1), REI supine baseline (41.4 vs. 49.9), SaO_2_ mean (92.5% vs. 91.9%), SaO_2_ nadir (77.6% vs. 77.2%), overjet (2.5 mm vs. 2.8 mm), overbite (3.0 mm vs. 3.0 mm) or maximum mouth opening (51.2 mm vs. 51.6 mm).

## 4 Discussion

To our knowledge, this is the first randomised crossover trial comparing OA treatment with and without elastic bands, based on an appropriate sample size estimation. Overall, the application of elastic bands significantly improved OA treatment success in this study. However, several subjective variables came out in favour of OA treatment without elastic bands.

The main benefit of applying elastic bands was a greater reduction in REI when sleeping in supine position. REI nonsupine did not differ between the two treatment modalities (Table 2). POSA has previously been recognised as a predictive factor for success with OA treatment in several studies [32, 33]. However, the majority of studies that identified POSA as a predictor of success with OA treatment used OA designs that restricted mouth opening during sleep, such as monobloc appliances [32, 33]. Studies that did not identify POSA as a predictive factor for OA success tend to use bibloc OAs that allow mouth opening to a greater extent, such as those used in the present study [15, 31].

Opening of the mouth with an OA in situ has been shown to increase pharyngeal collapsibility [18, 34], consequently leading to less effective OA treatment [35]. This mechanism is more pronounced in supine position due to gravity [36]. A recent study comparing a monobloc appliance with a bibloc appliance with elastic bands showed significantly better treatment effect in the bibloc group [37]. This finding indicates that the application of elastic bands on bibloc appliances enhances the positive functional mechanisms of the monobloc appliance, reducing the pharyngeal collapsibility. These results are supported by the findings in the current study. Hence, patients who might benefit from reduced mouth opening when treated with an OA should be considered for treatment with bibloc appliances with elastic bands rather than monoblocs for several reasons. In addition to superior treatment effect, biblocs are generally simpler to manufacture and titrate, and biblocs with elastic bands can easily be converted into a standard bibloc if issues regarding adherence or other problems occur.

A larger maximum mouth opening was associated with success exclusively with elastic bands. OAs that allow involuntary mouth opening exceeding the vertical range of the appliance might impair treatment efficacy during these episodes of sleep. Consequently, the appliance wings no longer hold the mandible in the desired protruded position leading to loss of pharyngeal patency. Hence, patients with large maximum mouth opening capacity should be considered for OA with elastic bands to prevent ‘losing’ the mandibular protrusive position.

A significantly greater chance of success with OA was observed for men when treated with elastic bands compared to women in the present study. This finding can be compared with a previous study evaluating the treatment effect of a monobloc appliance with nasal CPAP [38]. All patients included were men with moderate OSA, in addition to being categorised with POSA. The study showed no significant differences in treatment effect between the monobloc and nasal CPAP, meaning that men with moderate OSA and POSA may achieve excellent treatment effects with an OA that restricts mouth opening. The previously mentioned study from Marklund [33], using a monobloc device, identified POSA as a predictive factor for treatment success in men, but not in women. Similarly, reduction of supine AHI with elastic bands was significantly greater for men, but not for women, in the present study. This may indicate that men with POSA generally should be considered for OA designs that restrict mouth opening.

The frequency of reporting ‘improved quality of life’ during OA treatment was significantly higher without elastic bands. In addition, frequency of participants with excessive daytime sleepiness (ESS > 10) was significantly lower without elastic bands, as well as significantly higher frequency of reported usage ≥ 5 nights/week, compared to OA with elastic bands. These findings may indicate that the patients who completed this study preferred OA treatment without elastic band as a group. The fact that five patients dropped out due to nonadherence with elastic bands emphasises this. Previous studies on subjective outcomes of OA treatment comparing different OA designs did not identify a superior modality in this regard [39]. Furthermore, studies on patient preferences comparing OAs with monobloc design to bibloc design have conflicting results: Some studies show higher preferences towards bibloc designs [10, 11], whereas other comparable studies are in favour of monobloc designs [12, 40]. However, in the subgroup analysis, investigating differences between above‐mentioned subjective variables for men with large maximum mouth opening, no significant differences were observed. This indicates that with appropriate patient selection, the use of elastic bands may not affect patient preferences.

There is seemingly not one single OA design that is selected by all patients, and it seems necessary to individualise OA design per patient to increase both treatment effect and adherence. Therefore, future research needs to emphasise patient preferences to identify variables that may predict this on an individual level, in addition to investigating variables predicting treatment efficacy.

The main limitation of our study is the number of dropouts during the intervention. A total of 52 patients completed both interventions, meaning that the number of patients determined through sample size estimation (*n* = 62) was not reached due to considerably higher attrition rate than expected. Still, a significant difference in the primary outcome measure was observed, and the dropouts did not differ significantly from the patients completing both interventions in any of the investigated variables. However, variables predicting treatment success with elastic bands, such as male gender and large maximum mouth opening, cannot be generalised to apply for all OSA patients but need to be put in context with other similar studies before application. It should also be noted that some patients had missing data regarding REI nonsupine at baseline (Table 1), preventing the assessment of their POSA status. This resulted in a reduced sample size when investigating POSA as a predictive variable in the study population.

Furthermore, it was not possible to monitor objective adherence with the two treatment modalities because an adherence chip on the OAs was not included in the study. The rather short follow‐up time per treatment modality indicates an initial preference towards treatment without elastic bands, however, long‐term follow‐up studies are needed to investigate how elastic bands may affect adherence to OA treatment over time.

Due to the clinically implemented recruitment strategy in this study, patients meeting the inclusion criteria (being nonadherent to PAP) were consecutively invited by all clinicians performing follow‐up controls on their PAP therapy. Hence, it was not possible to estimate the exact number of patients who were assessed for eligibility. In addition, patients who are nonadherent to PAP may possess personality traits that also make them prone to be nonadherent to OA. Probably, an ‘unselected’ group of patients would have been preferable, although the study design reflects guidelines and the practice in most sleep centres internationally, selecting PAP as the primary choice of treatment. We did not apply a wash‐out period between the two treatment modalities due to ethical considerations, meaning we found it difficult to argue that patients deemed in need of treatment for moderate or severe sleep apnoea should be advised to discontinue treatment for a given period. Furthermore, we assumed no or limited carry‐over effect between the two treatment modalities, and we therefore did not consider a wash‐out period as obligatory.

The strength of this study is primarily its design. A randomised crossover trial is an optimal environment for comparing two treatment modalities, where neither has any significant persisting effects, eliminating most confounding factors which could be present in other randomised controlled trials with unmatched groups. In addition to this, we investigated the effect of OA treatment on several subjective variables using validated questionnaires, such as ESS, BIS, FSS 7‐item and HADS, which have been lacking in previous reports on the same theme. Furthermore, the study population was highly representative of noncompliant patients referred for OA treatment, as the patients were recruited in a clinical environment with relatively wide inclusion criteria. Hence, the results of this study are easily applicable in clinical practice.

## 5 Conclusion

The application of elastic bands to restrict mouth opening in OA treatment significantly improved treatment success compared to OA treatment without elastic bands (53.9% vs. 34.6%). These results apply to OA treatment using appliances that allow mouth opening. Patient groups that seemed to benefit from the application of elastic bands were men with large maximum mouth opening, reducing the REI in supine position. However, patients in the current study preferred OA treatment without elastic bands, emphasising the necessity of selecting patients who will benefit from them, rather than universally applying elastic bands on all OAs.

## Author Contributions

All authors contributed to concept and study design. U.L.O. collected the data. U.L.O., M.B. and A.J. analysed the data. S.L. and B.B. interpreted the data. U.L.O. wrote the paper. M.B., S.L., B.B. and A.J. revised the paper. All authors approved the final draft of the manuscript before submission.

## Conflicts of Interest

The authors declare no conflicts of interest.

### Peer Review

The peer review history for this article is available at https://www.webofscience.com/api/gateway/wos/peer‐review/10.1111/joor.13870.

## Data Availability

The data that support the findings of this study are available from the corresponding author upon reasonable request.
